# Outcomes and Predictors of Recurrence and Survival in Surgically Resected Localized Chromophobe Renal Cell Carcinoma: Results from the Canadian Kidney Cancer Information System (CKCis)

**DOI:** 10.3390/curroncol33030175

**Published:** 2026-03-19

**Authors:** Erica Arenovich, Rodney Breau, Ricardo Rendon, Ranjeeta Mallick, Simon Tanguay, Frederic Pouliot, Luke Lavallee, Andrew Feifer, Antonio Finelli, Rahul Bansal, Jean-Baptiste Lattouf, Miles Mannas, Bimal Bhindi, Jasmir G. Nayak, Naveen Basappa, Daniel Y. C. Heng, Aly-Khan A. Lalani, Georg Bjarnason, Lori Wood

**Affiliations:** 1 Queen Elizabeth II Health Sciences Centre, Halifax, NS B3H 3A7, Canada; 2 The Ottawa Hospital Research Institute, Ottawa, ON K1H 8L6, Canada; 3 Department of Urology, Dalhousie University, Halifax, NS B3H 4R2, Canada; 4 McGill University Health Centre, Montréal, QC H4A 3J1, Canada; 5 Division of Urology, CHU de Québec-Université Laval, Québec City, QC G1V 4G2, Canada; 6 Trillium Health Partners, Mississauga, ON L5M 2N1, Canada; 7 Department of Surgery, McMaster University, Hamilton, ON L8S 4L8, Canada; 8 Centre Hospitalier de l’Université de Montréal, Montréal, QC H2X 0C1, Canada; 9 BC Cancer Agency, Vancouver, BC V5Z 4E6, Canada; 10 University of Calgary, Calgary, AB T2N 1N4, Canada; 11 Department of Surgery, University of Manitoba, Winnipeg, MB R3T 2N2, Canada; 12 Department of Oncology, University of Alberta, Edmonton, AB T6G 2R3, Canada; 13 Juravinski Cancer Centre, Hamilton, ON L8V 5C2, Canada; 14 Sunnybrook Odette Cancer Center, Toronto, ON M4N 3M5, Canada

**Keywords:** chromophobe, renal cell carcinoma, outcomes, localized

## Abstract

Chromophobe RCC (chRCC) represents 5–10% of all RCC; however, data regarding outcomes and predictors of recurrence and survival in localized disease are limited. The Canadian Kidney Cancer Information System (CKCis) is a multi-institutional prospective cohort. Patients who had surgical resection for clinically localized chRCC between January 2011 and July 2024 were included. Descriptive statistics were used, and cancer recurrence and time to death were estimated using Kaplan–Meier curves. Associations between baseline and tumour characteristics and recurrence and survival were assessed using Cox proportional hazards models. The study cohort included 790 patients. Median follow-up was 4.9 years, mean age was 57.8 years and 57.5% were male. Partial nephrectomy occurred in 52.7% and radical in 47.3%. Recurrence-free survival was 93.6% at 5 years and 90.2% at 10 years. In the 45 patients who recurred, 40 had metastatic disease, 3 had local recurrences, and 2 had new contralateral disease. Predictors of recurrence included a higher pT stage, sarcomatoid features, positive margins, and tumour necrosis. Overall survival was 94.5% at 5 years and 83.7% at 10 years. Predictors of death included high pT stage, increase in pathological size, and increasing age at diagnosis. In this large Canadian cohort, patients with surgically resected, localized chRCC had favourable 5- and 10-year oncologic outcomes. These favourable outcomes are a reminder that not all RCC patients with non-clear cell histologies should be pooled together as a single entity. There is, however, a subgroup of patients with less favourable outcomes who should be the focus of future research that aims to prevent recurrence and RCC death.

## Simple Summary

Chromophobe renal cell cancer (chRCC) is an uncommon subtype of kidney cancer. This research focuses on patients who present with chRCC in their kidney and have it surgically removed. Baseline factors like sex, age, and size of tumour will be presented as well as pathological features of the cancer after surgical resection. This study shows that the majority of patients have very good outcomes, and cancer does not recur. There is, however, a subset of patients with more advanced disease in the kidney (higher stage and larger tumours) and other pathological features who have worse outcomes. Identifying these patients may help improve cancer outcomes in the future.

## 1. Introduction

Chromophobe renal cell carcinoma (chRCC) is the third most common histologic subtype of RCC, accounting for approximately 5–10% of RCC cases [1]. ChRCC tumours originate from the intercalated cells of the collecting duct and do not have the characteristic lipid and glycogen that clear cell RCCs have in their cytoplasm. chRCCs are not associated with the 3p chromosome deletion seen in clear cell RCC. TCGA studies show a relatively low mutational burden, with the only identified recurring alterations being TP53 and PTEN mutations [2].

The clinical data that does exist suggests patients with chRCC experience better outcomes compared to other RCC histologies [3,4,5,6,7,8]. However, given the relative rarity of chRCC, many trials and RCC observational studies have low patient numbers or are included in reports describing a heterogeneous group of all non-clear cell RCC histologies. Thus, our understanding of chRCC biology and outcomes is less robust than with clear cell RCC.

The Canadian Kidney Cancer Information System (CKCis) is a prospective cohort of patients from Canadian academic institutions. The large number of patients with detailed baseline and follow-up information allow for a nuanced description of rare RCC subtypes. In this manuscript we describe the baseline demographics and outcomes of chRCC patients diagnosed with clinically localized tumours and managed with surgical resection. Predictors of recurrence and survival will be explored.

## 2. Methods

### 2.1. Study Design

The CKCis cohort database was used to identify patients presenting with localized chRCC who had surgical resection between January 2011 and July 2024. Patients with synchronous metastatic disease were excluded. CKCis prospectively includes kidney cancer patients from 15 centres across Canada. It has been shown to be representative of the entire Canadian kidney cancer population [9]. All sites have Research Ethics Board approval for CKCis projects.

Information regarding baseline demographics, staging, laboratory and histology features, and surgical management were collected by trained data abstractors at each site. No strict follow-up schedules were prescribed, but generally, patients were followed with clinical assessments and imaging following the Canadian Urological Association Guidelines [10].

### 2.2. Outcomes

The primary outcome was cancer recurrence. Recurrence was classified as local recurrence or metastatic recurrence (or both). Patients with contralateral kidney tumour occurrence were documented but not considered a local recurrence. A secondary outcome was death from any cause.

### 2.3. Statistical Analysis

Baseline characteristics are presented as percentages for categorical variables and as means with standard deviation or medians with interquartile ranges for continuous variables, as appropriate.

Time-to-event outcomes were estimated using the Kaplan–Meier method. Time zero was the date of chRCC diagnosis. For recurrence, patients were censored at death from another cause or last follow-up. For death, patients were censored at last follow-up.

Cox proportional hazards regression models were used to evaluate the association between a priori selected patient and tumour characteristics and time-to-event outcomes. Variables included in the models were based on the prior published RCC literature and those with biological plausibility. In previously published reports, postoperative predictive models discriminate better than preoperative models, and thus, pathologic variables (such as pathologic stage; pT) were chosen preferentially over preoperative variables (such as clinical stage; cT) [11]. Variables included: pathological size of tumour (in cm), pT stage, presence of any sarcomatoid features, presence of any necrosis, and presence of a positive surgical margin. For overall survival (OS) modelling, Charlson Comorbidity Index score (CCI) and age were also included. CCI increased in 1-point increments and age at diagnosis increased in 1-year increments. CCI excluded the incident RCC diagnosis (1 point) and age (as age was included as a distinct variable).

For recurrence-free survival (RFS) modelling, patients were excluded if they presented with bilateral or multiple ipsilateral renal tumours, as they may inherently have a different biology compared to sporadic chRCC. Patients with pathologic lymph node metastases were excluded, as these small numbers of patients had occult metastatic disease. Contralateral kidney tumour occurrence was not considered in the recurrence analysis.

For overall survival (OS) modelling, all patients were included.

All statistical tests were two-sided with *p* < 0.05 considered statistically significant. All analyses were performed using the Statistical Analysis System Software (v9; SAS Institute, Cary, NC, USA).

## 3. Results

**Figure 1:**
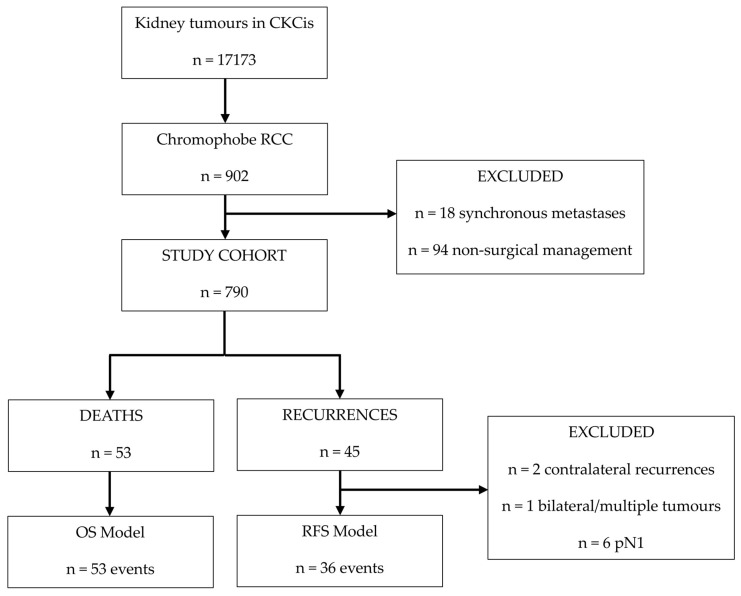
Consort diagram.

Among 17,173 patients in the CKCis cohort with benign and malignant kidney tumours, 902 had histologically proven chRCC. Patients with synchronous metastatic disease, n = 18 (2%), or non-surgical management, n = 94 (10.4%), were excluded. The final study cohort included 790 patients with clinically localized, surgically resected chRCC as shown in Figure 1. The median follow-up was 4.9 years (IQR = 2.2 to 7.4).

Baseline demographics, clinical staging, and pathological findings are shown in Table 1. The mean age at diagnosis was 57.8 years (SD = 12.9) and 454 patients (57.5%) were male. A single renal tumour was found in 766 (97.2%) patients. The mean imaging size of the largest primary tumour at diagnosis was 5.7 cm (SD = 3.9; range 0.7–30 cm). The cT stage was cT1 in 528 (70.2%), cT2 in 155 (20.6%), cT3 in 66 (8.8%), cT4 in 3 patients and missing in 38 patients. Enlarged lymph nodes (cN1) were observed in 15 (2.0%) patients.

Partial nephrectomy was performed in 416 (52.7%) patients and radical nephrectomy in 373 (47.3%). Surgery was laparoscopic in 397 (50.7%) patients, open in 267 (34.1%), and robotic in 119 (15.2%). The pT stage was pT1 in 495 (63.1%) patients, pT2 in 112 (14.3%), pT3 in 175 (22.3%), pT4 in 3 (0.4%), and missing in 5 patients. Tumour necrosis was present in 155 (20.1%) patients, positive margins were present in 58 (7.7%), and sarcomatoid features were noted in 12 (1.6%). Pathological node-positive disease (pN1) was recorded in eight (1.0%) patients.

### 3.1. Recurrence

**Figure 2:**
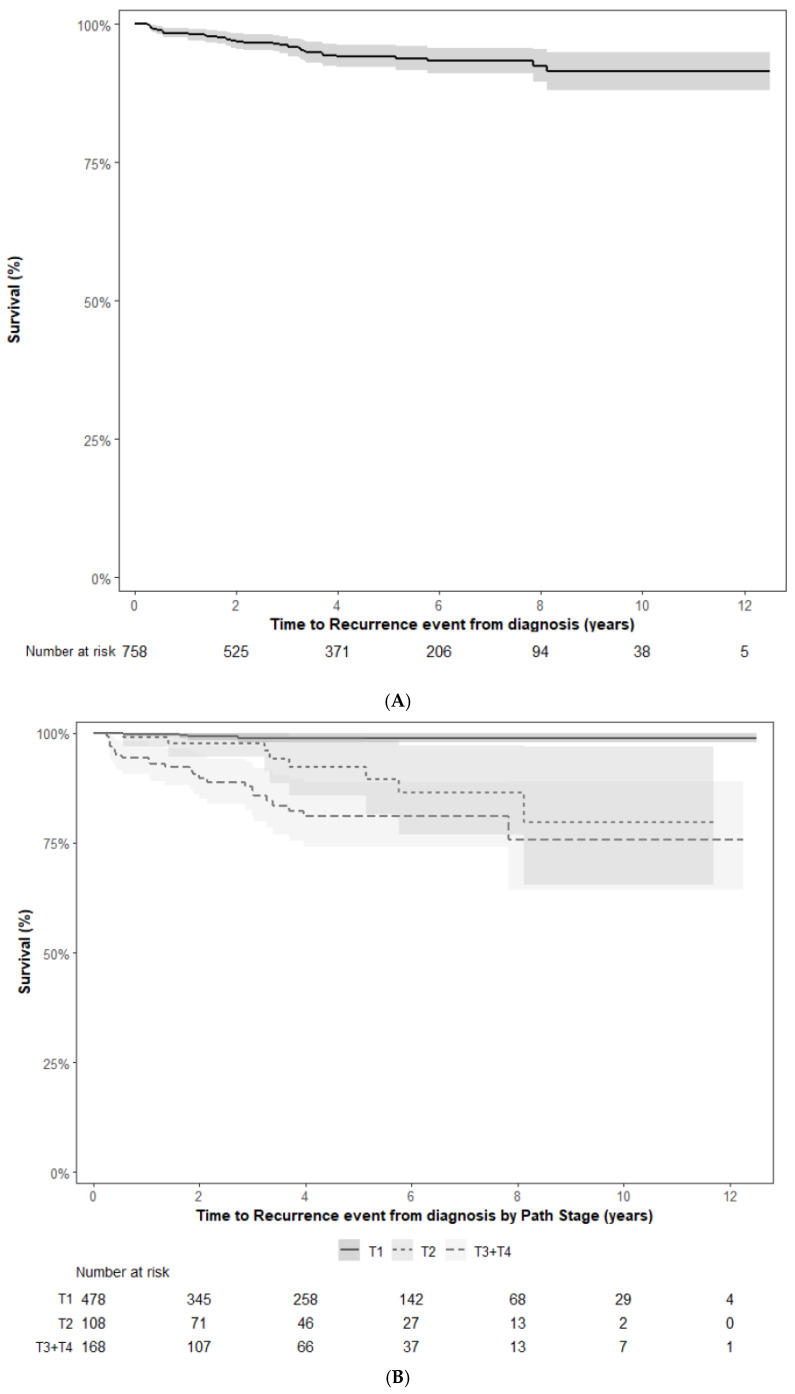
(**A**) Recurrence-free survival (in years with 95% confidence intervals). (**B**) Recurrence-free survival stratified by pT stage (in years with 95% confidence intervals).

The 2-, 5- and 10-year recurrence-free survival for this cohort is 96.6%, 93.6% and 90.2% respectively as shown in Figure 2A. In the 45 patients who recurred, 2 patients had new contralateral primaries, 3 patients had local recurrences, and 40 patients had metastatic disease.

At the time of recurrence, the two patients with contralateral kidney recurrences were treated with local therapy (surgery and radio-frequency ablation) and remained disease-free at last follow-up (8.2 and 5.4 years). The three patients with local recurrences were managed by radio-frequency ablation at the prior partial nephrectomy site, metastasectomy of a renal bed lesion, and stereotactic body radiation therapy to a retroperitoneal soft tissue mass. Of the remaining 40 patients with metastatic recurrence, the first management included systemic therapy in 19 (47.5%), metastasectomy in 11 (27.5%), surveillance in 5 (12.5%), radiation in 4 (10%), and no treatment in 1 patient due to comorbidities. In these 40 patients, at least one line of systemic therapy was utilized in 29 patients (72.5%) and radiation in 21 (52.5%) patients.

### 3.2. Predictors of Recurrence

The a priori variables included in the RFS model for pN0 solitary chRCC patients are shown in Table 2. Predictors of recurrence include pT3/T4 vs. pT1 disease (HR: 12.20; CI: 2.66–56.07; *p* value: 0.001), pT2 vs. pT1 disease (HR: 5.36; CI: 1.12–25.60; *p* value: 0.035), the presence of sarcomatoid features (HR: 7.08; CI: 1.94–25.77; *p* value: 0.003), a positive surgical margin (HR: 4.21; CI: 1.79–9.88; *p* value: 0.001), and the presence of necrosis (HR: 3.69; CI: 1.65–8.23; *p* value: 0.001).

As pT stage was the most significant variable for recurrence, RFS stratified by pT stage is shown in Figure 2B. 

### 3.3. Survival

**Figure 3:**
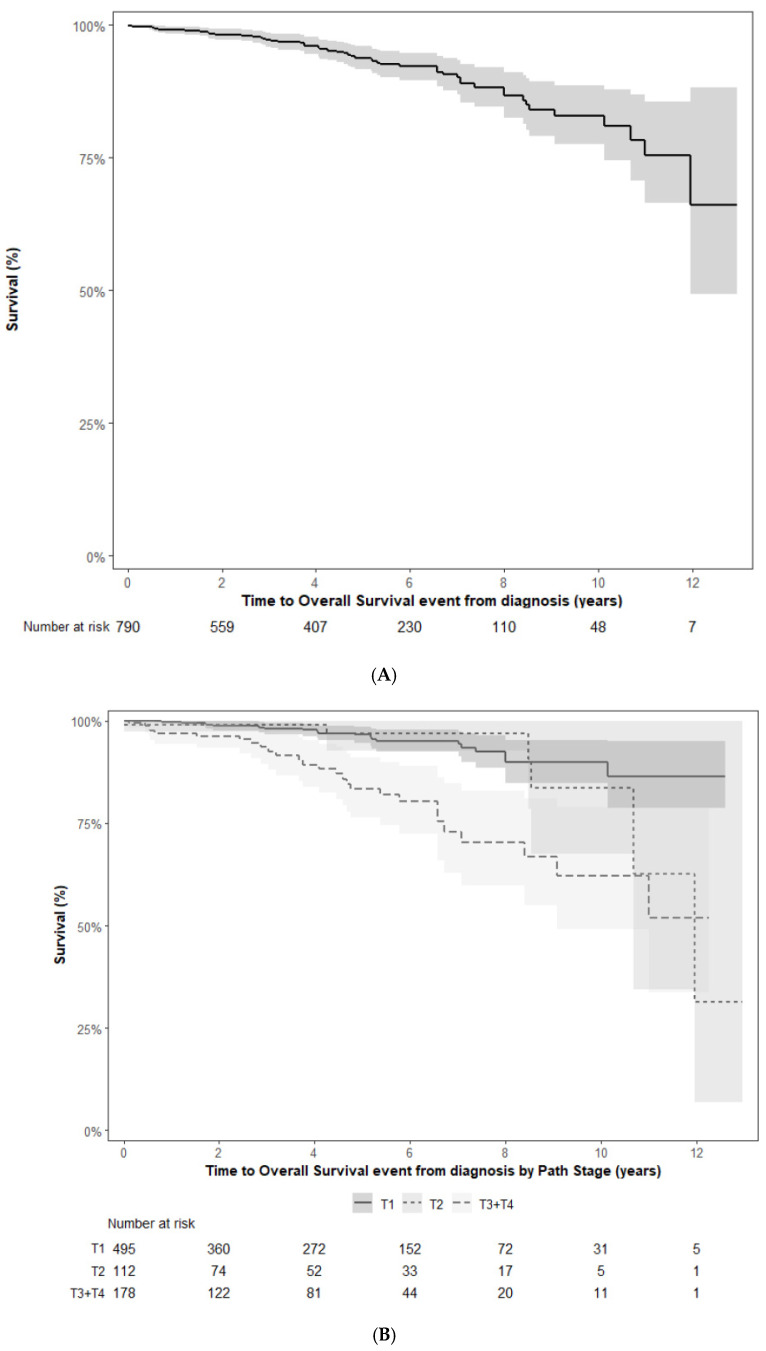
(**A**) Overall survival (in years with 95% confidence intervals). (**B**) Overall survival stratified by pT stage (in years with 95% confidence intervals).

The 2-, 5- and 10-year overall survival was 98.8%, 94.5% and 83.7% respectively as shown in Figure 3A. During follow-up, 53 patients died with the cause of death being RCC in 20 (37.7%), non-RCC cancer in 10 (18.9%), non-cancer death in 7 (13.2%) and unknown in 16 (30.2%).

### 3.4. Predictors of Overall Survival

The a priori variables included in the OS model are shown in Table 2. Predictors of worse survival from any cause included pT3/T4 vs. pT1 disease (HR: 3.14; CI: 1.58–6.23; *p* value: 0.0011), increase in pathological primary tumour size (HR: 1.12; CI: 1.01–1.24; *p* value: 0.040), and increasing age at diagnosis as a continuous variable (HR: 1.09; CI: 1.04–1.15; *p* value: 0.0001). Given that both pathological tumour size and pT stage were significant, sensitivity analyses were performed removing each of these variables and the results are consistent.

As pT stage was the most significant variable for survival, OS stratified by pT stage is shown in Figure 3B.

## 4. Discussion

In this study, we analyzed a large cohort of patients presenting with localized chRCC treated with surgical resection managed at CKCis-participating Canadian cancer centres. chRCC is an uncommon histological subtype and is often lumped into studies reporting heterogeneous groups of non-clear cell RCC; however, given its unique histological, molecular, and clinical features, it should be reported as its own entity [4,12]. This research involving a real-world cohort of localized chRCC patients contributes to the existing body of knowledge.

Compared to other RCC histological subtypes, chRCC appears to have more favourable outcomes. Many patients with localized chRCC do not recur, with the 5- and 10-year RFS being 94% and 90% respectively in our cohort. Casuscelli et al. have published on a cohort of localized chRCC patients (n = 496) treated surgically at Memorial Sloan Kettering Cancer Center (MSKCC) between 1990 and 2016 [13]. Their 5- and 10-year RFS was 94.9% and 91.7% respectively.

In the MSKCC report, the 5- and 10-year OS rates for chRCC patients were 92.3% and 82.1%, respectively, compared to 81.7% and 63.6% of patients with clear cell RCC (n = 3312). In another publication using National Cancer Database data, the 5-year OS was 91% for chRCC patients (n = 12,760) compared to 82% in papillary (n = 27,510) and 81% in clear cell RCC (n = 178,066) [3]. The 5- and 10-year overall survival of 95% and 84% in our cohort is very similar to these prior reports although our 10-year outcomes must be interpreted with the knowledge that our median follow-up was 5 years. It should also be noted that many of the patients in our cohort died of causes unrelated to RCC.

Despite several recognized models and nomograms that predict post-surgical outcomes in localized RCC, none are chRCC-specific. Existing models primarily focus on clear cell RCC using different variables including age, tumour size, clinical presentation, pathological size, stage, grade, necrosis, and lymph node status [14,15,16,17,18,19,20,21,22]. To note, Fuhrman grade is not applicable to chRCC. The Kattan, UCLA UISS, and ASSURE models include patients with chRCC, but they make up a small proportion: 10.4% in the Kattan data, an unknown percentage in the UCLA UISS data, and 5.5% in the ASSURE data. Interestingly, in the ASSURE model, chromophobe and papillary type I RCC were combined and used as the reference histology and had better outcomes compared to other histologies (predominantly clear cell).

Our RFS model included pN0 patients with solitary tumours. The pN1 patients were not included as six out of eight of those patients recurred, indicating that it is a very strong predictor of recurrence and that including them would create instability in the model due to the effect size. This population of pN1 patients likely represents an extremely aggressive subset of chRCC. The most significant variable associated with a higher risk of recurrence in our study was higher pT stage, especially pT3/T4 disease. Patients with pT3/T4 disease versus pT1 had a 12 times higher risk of recurrence and those with pT2 versus pT1 disease had a 5 times higher risk of recurrence. The presence of sarcomatoid features was also a very significant predictor with a 7 times higher risk of recurrence. Sarcomatoid features were noted in 1.6% of our cohort but present in 15.7% of patients who recurred. The presence of sarcomatoid features in chRCC varies in the literature from 1.8% to 9% and has been previously shown to be a predictor of worse outcomes. [23,24]. The presence of rhabdoid features may also be a predictor of worse outcomes and warrants further study; however, it was not included as a variable in our study as it has only recently been added to the CKCis database. The presence of necrosis and positive surgical margins were also predictors of recurrence. Tumour necrosis was present in 20% of our patients and varies from 7.3% to 36% in the literature and has also been shown to predict worse outcomes [13,25,26].

In our OS model, predictors of worse survival included larger pT stage, an increase in pathological size, and increased age at diagnosis. Patients with sarcomatoid features had a 3-fold increase in risk of death; however, this was not statistically significant.

To date, no chRCC-specific postoperative surveillance guidelines exist [10,27,28,29,30,31]. Most guidelines focus on clear cell RCC and incorporate pT and pN status or stage and some incorporate Fuhrman grade. Only the American Urological Association guideline specifically incorporates sarcomatoid and rhabdoid features and always places these patients in the very-high-risk group. The European Association of Urology guideline [30] is the only one to separate out non-clear cell RCC; however, once again, all non-clear cell histologies are lumped together. No guideline explicitly incorporates the presence of necrosis or positive surgical margins, which were found to be significant predictors of recurrence in our study. Thus, until a chRCC-specific follow-up guideline exists, utilizing one that offers a more intensive follow-up for the highest risk chRCC patients may be the most appropriate. Our results also suggest there may be a subset of resected chRCC patients who have a very low risk of recurrence or death and for whom follow-up may be de-escalated. Further research on this very-low-risk group is warranted.

To date, there are no published trials to support the use of adjuvant therapy even in the highest risk resected chRCC patients as most adjuvant clinical trials enrolled only clear cell RCC [32]. A few older trials did allow non-clear cell RCC patients, but they made up small numbers (5.5% in ASSURE and 6.4% in EVEREST) [33,34]. The positive adjuvant trial with pembrolizumab did not include non-clear cell RCC patients [35]. The RAMPART trial recently reported a 3-year disease free survival benefit for high-risk patients with 16% non-clear cell RCC patients enrolled and may offer further insights [36]. Despite our study identifying a subset of chRCC patients at the highest risk of recurrence, conducting randomized trials to examine the role of adjuvant therapy in these patients is impractical due to the low event rate and thus the large sample size that would be needed. Also, the optimal systemic therapy for chRCC is still unknown. Currently, there is no evidence-based role for adjuvant systemic therapy in chRCC.

The largest strength of this study is that it includes a large cohort of chRCC patients managed in multiple centres across several provinces and varied health care centres and thus is generalizable to the real-world setting. A major limitation includes the lack of a centralized pathology review to confirm chRCC as well as other pathological findings like the presence of sarcomatoid features. Other limitations relate to the low number of events in our study. This resulted in the inability to perform an assessment of the proportional hazard assumption. It also resulted in wide confidence intervals for some of the significant variables in our RFS and OS models. Also, studying the impact of post-recurrence treatments and their association with survival was beyond the scope of this study but is the focus of ongoing CKCis research. Finally, as with many real-world databases, some data is missing including cause of death in some patients, which prevented us from performing a competing risk analysis.

## 5. Conclusions

This large Canadian cohort of patients treated with surgical resection for localized chRCC showed favourable outcomes in terms of recurrence-free and overall survival. These outcomes offer a reminder that RCC studies must be conducted and reported for individual histologies, rather than as a larger cohort of non-clear cell RCC patients. There is a subgroup of chRCC patients with less favourable outcomes who should be the focus of future research that aims to prevent recurrence and RCC death.

## Figures and Tables

**Table 1:** Demographic, clinical and pathological findings.

	n = 790
Age at Diagnosis, Years, Mean and SD	57.8 (12.9)
Sex, n (%)	
Male	454 (57.5)
Female	336 (42.5)
Imaging Size, Mean (Range in cm)	5.7 (0.7–30)
CCI, n (%)	(n = 742) *
2	130 (17.5)
3–4	353 (47.6)
≥5	259 (34.9)
Clinical T Stage, n (%)	(n = 752) ^#^
cT1	528 (70.2)
cT2	155 (20.6)
cT3	66 (8.8)
cT4	3 (0.4)
Clinical N Stage, n (%)	(n = 753) ^^^
N1	15 (2.0)
Pathological Stage, n (%)	(n = 785) ^!^
pT1	495 (63.1)
pT2	112 (14.3)
pT3	175 (22.3)
pT4	3 (0.4)
pN1	8 (1.0)
Pathological Findings, n (%)	
Necrosis	155 (20.1) **
Sarcomatoid Features	12 (1.6) ^##^
Positive Margins	58 (7.7) ^^^^

SD: Standard Deviation, CCI: Charlson Comorbidity Index. * missing data in 48, ^#^ missing data in 38, ^^^ missing data in 37, ^!^ missing data in 5, ** missing data in 20, ^##^ missing data in 21, and ^^^^ missing data in 34 patients.

**Table 2:** Predictors of recurrence and survival in multivariate analysis.

	Hazard Ratio for RFS (95% CI)	*p*-Value	Hazard Ratio for OS (95% CI)	*p*-Value
Increase in Pathological Size (cm)	1.03 (0.94–1.14)	0.5	1.12 (1.01–1.24)	**0.040**
pT2 vs. pT1	5.36 (1.12–25.60)	**0.035**	0.94 (0.29–3.01)	0.92
pT3/T4 vs. pT1	12.20 (2.66–56.07)	**0.0013**	3.14 (1.58–6.23)	**0.0011**
Presence of Necrosis	3.69 (1.65–8.23)	**0.0014**	0.98 (0.49–1.97)	0.96
Presence of Sarcomatoid	7.08 (1.94–25.77)	**0.003**	2.93 (0.62–13.83)	0.17
Presence of Positive Margin	4.21 (1.79–9.88)	**0.001**	2.01 (0.71–5.73)	0.19
Increase in Age by 1 year			1.09 (1.04–1.15)	**0.0001**
Increase in CCI by 1 point			1.16 (0.99–1.35)	0.061

CI: confidence interval, CCI: Charlson Comorbidity Index, vs.: versus, RFS: recurrence-free survival, and OS: overall survival. The bold represents statistically significant difference in the analysis.

## Data Availability

Data is unavailable due to privacy concerns, restricted data transfer of agreements, governance regulations, and specific Research Ethics Board restrictions.
